# A Wearable System with Embedded Conductive Textiles and an IMU for Unobtrusive Cardio-Respiratory Monitoring

**DOI:** 10.3390/s21093018

**Published:** 2021-04-25

**Authors:** Joshua Di Tocco, Luigi Raiano, Riccardo Sabbadini, Carlo Massaroni, Domenico Formica, Emiliano Schena

**Affiliations:** 1 Unit of Measurements and Biomedical Instrumentation, Università Campus Bio-Medico di Roma, Via Alvaro del Portillo, 00128 Rome, Italy; r.sabbadini@unicampus.it (R.S.); c.massaroni@unicampus.it (C.M.); e.schena@unicampus.it (E.S.); 2 Unit of Neurophysiology and Neuroengineering of HumanTechnology Interaction (NeXT), Università Campus Bio-Medico di Roma, Via Alvaro del Portillo, 00128 Rome, Italy; l.raiano@unicampus.it (L.R.); D.Formica@unicampus.it (D.F.)

**Keywords:** cardio-respiratory monitoring, wearable system, wearable device, smart textile, IMU, respiratory rate, heart rate

## Abstract

The continuous and simultaneous monitoring of physiological parameters represents a key aspect in clinical environments, remote monitoring and occupational settings. In this regard, respiratory rate (RR) and heart rate (HR) are correlated with several physiological and pathological conditions of the patients/workers, and with environmental stressors. In this work, we present and validate a wearable device for the continuous monitoring of such parameters. The proposed system embeds four conductive sensors located on the user’s chest which allow retrieving the breathing activity through their deformation induced during cyclic expansion and contraction of the rib cage. For monitoring HR we used an embedded IMU located on the left side of the chest wall. We compared the proposed device in terms of estimating HR and RR against a reference system in three scenarios: sitting, standing and supine. The proposed system reliably estimated both RR and HR, showing low error averaged along subjects in all scenarios. This is the first study focused on the feasibility assessment of a wearable system based on a multi-sensor configuration (i.e., conductive sensors and IMU) for RR and HR monitoring. The promising results encourage the application of this approach in clinical and occupational settings.

## 1. Introduction

Continuous, real-time and non-invasive monitoring of vital signs through wearable devices represents one of the most appealing challenges posed by the modern medicine, healthcare and occupational health [1,2]. Regarding modern medicine and healthcare, the use of unobtrusive, lightweight and comfortable wearable devices for collecting physiological signals constitutes a key aspect for improving both the monitoring in clinical settings and a remote/home monitoring of the patients [3]. In clinical settings, a continuous monitoring becomes challenging in all those wards hospitalizing patients which require particular care because they have to be connected to bulky, portable, monitoring devices and every movement around the hospital becomes thus difficult [3,4]. Outside the clinic, wearable devices have gained increased attention for the remote monitoring of the patients and healthcare, due to their intrinsic comfortably, ease of use and reduced costs [3,5,6,7]. Moreover, the use of wearables to monitor physiological parameters has gained attention in occupational health as well, due to the increased attention to the workers’ health and safety by monitoring their condition in the era of Industry 4.0 [8]. Indeed, the monitoring of physiological parameters is beneficial to assessing physiological status, and the activities and fatigue levels of workers (e.g., muscle-skeletal and cardiovascular disorders) to improving their health, well-being and safety and thus meeting the guidelines defined by ergonomics [6,9,10].

In these scenarios, respiratory rate (RR) and heart rate (HR) have gained broad interest, since they are strictly related to different physiological and pathological conditions of the patients/workers (e.g., early detection of critical events) and to different environmental stressors [6,11,12]. These vital signs can be monitored using many approaches [9,13].

In this work, we present a prototype of a novel wearable device for simultaneous monitoring of the cardio-respiratory parameters (i.e., RR and HR). The proposed system uses different sensors with respect to what has been reported in the literature and used in commercial devices, since it is based on four conductive textiles (for RR monitoring) and an IMU (for monitoring HR). These sensors were embedded within a highly integrated, lightweight, comfortable and low-cost wearable device. We have tested the feasibility of the proposed device in three different scenarios to mimic conditions that can be experienced in the above-described fields. Specifically, we enrolled eight healthy volunteers and we monitored their cardio-respiratory activity, in terms of RR and HR estimation, in three different scenarios: (i) sitting (e.g., it can simulate the occupational settings of a computer worker), (ii) standing and (iii) supine position (e.g., they can simulate clinical and remote applications). This work is organized into the following sections: (i) in Section 2 we focus on the related works; (ii) in Section 3 and Section 4 we describe the proposed wearable system (WS) and the experimental protocol used to assess its feasibility in monitoring RR and HR; (iii) in Section 5 we describe the techniques of data analysis used to estimate RR and HR starting from the raw data recorded by the WS; (iv) Section 6 reports the results in terms of both RR and HR; (v) Section 7 deals with the discussion of the obtained results and the conclusion.

## 2. Related Works

The state of the art of wearable systems for RR monitoring consists of techniques based on the cyclic expansion and contraction of the rib cage during the breathing activity. Most of these systems directly measure the expansion of the rib cage by means of electrical elements that change their impedance with strain (i.e., resistive and piezoresistive sensors, capacitive sensors and inductive sensors) and fiber optic sensors [14,15,16,17,18,19,20]. Fiber optic sensors (e.g., fiber Bragg grating sensors) have some advantages over their electrical counterparts related to their metrological properties (high sensitivity, good accuracy and short response time), immunity from electromagnetic interference and small size, and they are most often used in this field [17,21,22,23,24]. However, the interrogation systems are usually bulky and only recently have there been commercially available portable systems, but these remain quite expensive solutions (from around 3.000 USD to 40.000 USD). When the application does not require the use of the system in a harsh environment in terms of electromagnetic field (e.g., patients monitoring during magnetic resonance scan [23,24]), the resistive, capacitive and inductive sensors may be valid alternatives due to the low prices of both the sensors and the front-end electronics, and the possibility to collect the data by wireless transmission protocol [25,26]. Among others, resistive sensors represent a convenient solution to implement reliable, accurate and low-cost assessments of breathing activity and RR [9,27]. In addition, they can be manufactured as "smart textiles"; thus, it is possible to design highly integrated solutions maximizing the comfort and minimizing the encumbrance of the system itself [28,29]. A commercially available solution for RR monitoring is the SS5LB by BIOPAC systems Inc., which transduces the chest wall deformations using a strain gauge. To allow the collection of the transduced signal, an additional component has to be purchased, increasing both the complexity and costs. Moreover, the device cannot be used in unstructured and unsupervised environments [30]. As regards HR, many techniques have been proposed to develop wearable devices. They are mainly based on electrocardiography (ECG), photoplethysmography (PPG) and the monitoring of the local mechanical vibrations provided by the heartbeat to the chest wall, in terms of accelerations (seismocardiography, SCG) [21,31] or local angular rotations (gyrocardiography, GCG) [32,33]. Specifically, monitoring the cardiac activity using chest wall induced vibrations is an appealing solution for developing highly integrated wearable systems due to the recent technological advancements that have been made in micro-electromechanical systems (MEMSs) for motion tracking that integrate tri-axial accelerometers and gyroscopes into a miniaturized inertial measurement unit (IMU) [31]. Available commercial devices for monitoring HR based on a PPG sensor proposed by Polar. Different devices have been developed to match the needs of subjects (i.e., humans and animals) when monitoring their HRs during physical activities [34,35]. One of the limitations of these devices which is crucial for the application of interest is the inability to simultaneously monitor RR and HR. There are several solutions for monitoring RR and HR by wearable systems; however, the state of the art of wearable systems for simultaneous monitoring of these two parameters consists only of a few works. In [36,37] the system was based on electrodes placed in contact with the subject’s skin to monitor both ECG and breathing-induced variations of chest wall impedance during the cyclic respiration. In [22] fiber optic sensors were used for the mentioned purpose. In [38] a piezoelectric sensor was adopted to monitor SCG and breathing activity. In [26] a wearable belt embedding a capacitive sensor and two conductive textiles used as electrodes for a single lead ECG were used to monitor RR and HR simultaneously. Although this system is compliant with the scenarios presented in this study, it is characterized by a high price and having no feature to cope with sensor damage or data loss due to the sensor’s failure.

## 3. Experimental Setup

### 3.1. Wearable Device

The wearable device, hereinafter referred to as *WS*, consists of two main components: the first one uses 2 elastic bands; the second one is a a custom electrical board. The elastic bands utilizes 2 sensing elements each. The sensing elements are conductive textiles laser-cut as rectangles (dimensions L × W 50 mm × 10 mm) from an A4 sheet of material (Eeontex LG-SLPA by Eeonyx Corporation). When these textiles undergo strain, their initial resistance changes according to the applied strain. In this case, the strain is provided by the expansion and contraction of the rib cage during ventilation. To retrieve the respiratory signal on the rib cage, the sensing elements are hand sewed into the elastic bands on the extremities with silver-coated yarn (mod.235/36 dtex 2-ply HC, Statex Produktions und Vertieb GmbH, Germany), whose purpose is twofold: (i) to fix the sensing element to the band and (ii) to provide the electrical contact to retrieve the sensor’s output signal by connecting it to the electronic board. In addition, the elastic bands are provided with Velcro to allow the adaptability of the system to different anthropometries.

The custom electrical board has two main functions:

- To process the signal retrieved by the four conductive sensors. To accomplish this task it has 4 embedded Wheatstone bridges (1/4 bridge configuration with the sensing element connected in series with a trimmer of 50 kΩ with the other resistances of 82.5 kΩ) to transduce the conductive sensors’ output (i.e., an electrical resistance) into a voltage, 2 instrumentation amplifiers (AD8426 by Analog Devices) with a set gain of 6 and a microcontroller (STM32F446RET by STMicroelectronics).
- To retrieve the cardiac activity information and the position of the subject by using a Magneto-Inertial Measuring Unit (M-IMU, LSM9DS1 by STMicroelectronics).

In addition, the board is equipped with a microSD card socket for storage the data related to respiratory activity (provided by the 4 conductive sensors) and to heart activity (provided by the IMU). All data were collected at 100 Hz. The electronics are powered by a 750 mAh Li-Po battery at 3.7 V, which guarantees autonomy of approximately 8 h. The electronic board along with the battery was placed into a custom 3D-printed TPU casing.

**Figure 1:**
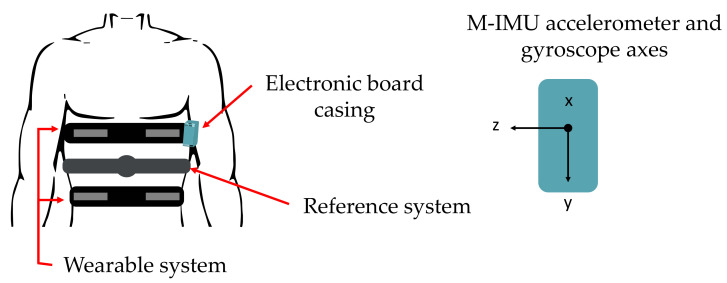
Schematic representations of the proposed wearable system and the reference system, and their positioning on the rib cage.

Figure 1 shows a schematic representation of the developed wearable system, the M-IMU axes’ orientation and the reference system.

The first step was to assess the response of the sensing elements by applying strain of up to 10%. We repeated 4 quasi-static trials and we calculated the calibration curve and the sensors’ sensitivity. The output of the sensors (an electrical resistance) was transduced in a voltage by a voltage divider (61.9 kΩ) powered at +5 V. Therefore, the calibration curve represents the relationship between the output of the amplification stage and the applied strain. It is the well represented by a second-order polynomial ($y=0.12\cdot x^{2}-3.81\cdot x+59.97$), as confirmed by the high value of the correlation coefficient ($R^{2}>0.99$).

### 3.2. Reference System

A reference system (Zephyr BioHarness 3.0 by Medtronic) provided the RR (collected at 25 Hz) and HR (single lead ElectroCardioGram, ECG, collected at 250 Hz).

## 4. Population and Experimental Protocol

To assess the performance of the proposed wearable system, we enrolled 8 healthy male volunteers (mean ± standard deviation: age—27.8 ± 2.7 years old; body mass—75.4 ± 12.2 kg; height—1.74 ± 0.08 m). Table 1 shows details regarding the subjects’ ages and somatotypes.

Informed consent was obtained from all subjects involved in the study (protocol code 27.2(18).20 of 15/06/2020), and the principles of declaration of Helsinki and amendments were followed in all the study’s steps.

Firstly, each volunteer was asked to wear the reference instrument belt on the xiphoid process line and the 2 elastic belts (one on the nipple line and one on the umbilical line). Both systems were worn in direct contact with the skin. The electronic board was positioned on the left side of the upper belt (next to the heart), in order to retrieve the cardiac activity displacements on the chest wall. Then, the volunteer was asked to perform approximately 10 s of self-paced breathing, a0 s apnea at the end of the inspiratory phase, 3 min of self-paced breathing and finally a 10 s apnea at the end of the inspiratory phase. The same protocol was applied in 3 different positions (i.e., standing, sitting and supine) for a total of 24 trials. The 9-axes M-IMU and the output of the 4 Wheatstone bridges along with the reference system parameters were collected simultaneously.

**Figure 2:**
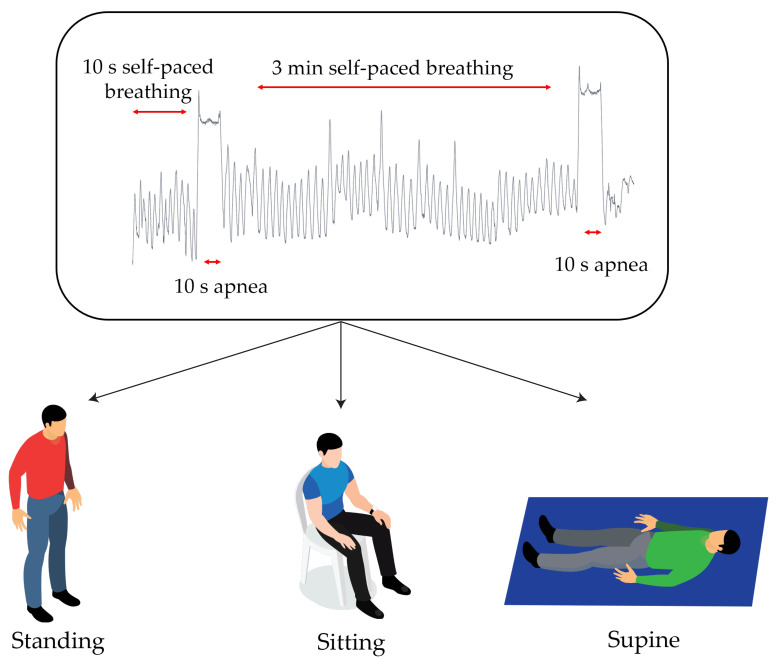
A schematic representation of the experimental protocol performed. The top trend represents the respiratory trial performed in the 3 tested scenarios shown in the lower part.

Figure 2 shows a graphical representation of the experimental setup and the protocols performed.

## 5. Data Analysis

The data analysis aimed at accomplishing two tasks: (i) estimating RR and HR starting from the trends of the conductive sensors’ output and from the IMU; (ii) assessing the performance of the proposed wearable system by comparing the values of RR and HR estimated by the wearable system and the reference one. In this regard, we implemented both a frequency domain analysis, for estimating average RR during the trials, and a time domain analysis to estimate RR breath-by-breath) [27].

To estimate HR we considered the signals recorded by the embedded IMU, and we analyzed them using two approaches: (i) we implemented a frequency domain analysis to monitor the average HR on the whole trial (it lasted approximately 3 min); (ii) a windowed frequency domain analysis considering windows of 30 s. This solution allows investigating how HR behaves over time.

The data analysis was entirely implemented in MATLAB^®^ for each subject and each protocol.

### 5.1. Respiratory Activity: Data Analysis

To assess RR we considered the signals recorded by the conductive sensors, which followed the breathing-related motions of the subjects’ rib cages (see Section 3.1). Specifically, we considered the average in time of the sensors of the four recorded conductive signals, hereinafter denoted as $r_{WS}\left(t\right)$. Both the conductive signal and reference signal ($r_{ref}\left(t\right)$) were filtered using a third-order Butterworth band-pass filter between 0.05 Hz and 2 Hz using zero-phase digital filtering implemented through the function "*filtfilt*" (embedded in MATLAB^®^). We selected 0.05 Hz as the low cut-off frequency in order to discard very slow signal variations from the recorded data; conversely, we selected 2 Hz as the high cut-off frequency, since RR is hardly above 1.5 Hz [9]. The choice of filtering the RR signals within the mentioned frequency band (i.e., from 0.05 to 2 Hz) agrees with the results reported in [27]. We aimed to filter out components not relevant for our applications while avoiding discarding any useful information recorded by the sensors [9,39].

#### 5.1.1. Frequency Domain Analysis

For the *i*-th subject we computed the error between the RR estimated using the spectrum of $r_{ref}\left(t\right)$ ($F_{ref,i}^{RR}$) and $r_{WS}\left(t\right)$ ($F_{WS,i}^{RR}$) in each scenario as follows:

(1) $${\tilde{F}}_{WS,i}^{RR}=\left|F_{ref,i}^{RR}-F_{WS,i}^{RR}\right|$$

$F_{ref}^{RR}$ and $F_{WS}^{RR}$ correspond to the highest peak in the spectra within the range 0.1–1.5 Hz. In (1) all terms are expressed in *bpm*, denoting breaths per minute. The spectra of the signals were obtained by computing the power spectral density (PSD) considering Welch’s overlapped segment averaging estimator over the duration of the trials (180 s). To that end, we used the MATLAB^®^ function "*pwelch*." In addition, we computed the percentage version of (1) as follows:

$${\tilde{F}}_{WS\%,i}^{RR}=\frac{|F_{ref,i}^{RR}-F_{WS,i}^{RR}|}{F_{ref,i}^{RR}}$$

The averages of subjects for ${\tilde{F}}_{WS,i}^{RR}$ and ${\tilde{F}}_{WS\%,i}^{RR}$ are denoted as ${\tilde{F}}_{WS}^{RR}$ and ${\tilde{F}}_{WS\%}^{RR}$, respectively.

#### 5.1.2. Time Domain Analysis

**Figure 3:**
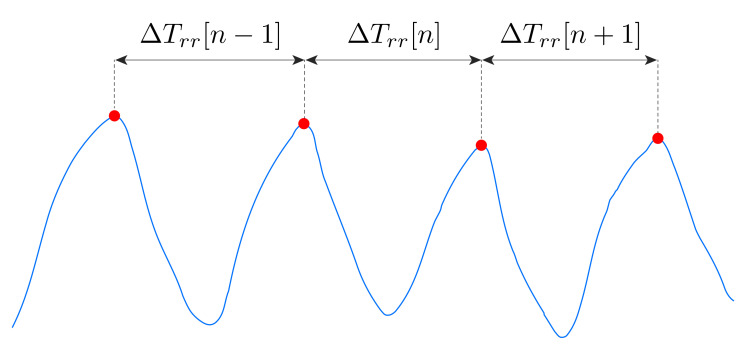
A schematic representation of the breathing act period $\Delta T_{rr}\left[n\right]$. The blue line represents $r_{WS}\left(t\right)$, and the red circles represent the identified respiratory peaks.

To implement a breath-by-breath analysis we computed the breath duration ($\Delta T_{rr}\left[n\right]$) between two inspiratory peaks both considering $r_{WS}\left(t\right)$ and $r_{ref}\left(t\right)$. To that end, we implemented the following steps [28]:

- The first step was devoted to the identification of the inspiratory peaks. We used the MATLAB^®^ function "*findpeaks*" with the inverse of the average RR (the value estimated using the frequency domain analysis) as temporal threshold; we used as amplitude threshold 50% of the *RMS* of $r_{WS}\left(t\right)$ during the entire duration of the task, and concerning $r_{ref}\left(t\right)$ we used as the amplitude threshold 40% of its RMS.
  We used two different amplitude thresholds to optimize the detection of the peaks. After this step, we visually inspected the detected peaks and eventually removed those not related to the end of inspiratory phase. This correction was performed on the data collected by the reference system and by the wearable system, mainly in the supine position, and it was needed due to the different morphologies of the signals which are affected by the position assumed by the subject.
- The second step was devoted to computing the period of each breathing act, $\Delta T_{rr}\left[n\right]$. This parameter was considered as the time elapsed between two consecutive peaks. This analysis was performed for both the wearable system ($\Delta T_{rr,WS}$) and the reference one ($\Delta T_{rr,ref}$) (see Figure 3);
- The third step was devoted to computing the RR for the *n*-th breath as $\frac{60}{\Delta T_{rr}\left[n\right]}$, for $r_{WS}\left(t\right)$ ($f_{WS}^{RR}\left[n\right]$) and $r_{ref}\left(t\right)$ ($f_{ref}^{RR}\left[n\right]$).

To compare the *WS* with the reference system in the time domain for the *i*-th subject and each protocol, we computed the mean absolute error ($MAE_{WS,i}$) as follows:

(2) $$MAE_{WS,i}=\frac{1}{N_{breaths}}\sum_{n=1}^{N_{breaths}}\left|f_{ref}^{RR}\left[n\right]-f_{WS}^{RR}\left[n\right]\right|$$

In (2), $N_{breaths}$ denotes the number of breaths identified in the *i*-th subject and the specific scenario. In addition, we computed the percentage version of (2) as follows:

$$MAE_{WS\%,i}=\frac{1}{N_{breaths}}\sum_{n=1}^{N_{breaths}}\frac{|f_{ref}^{RR}\left[n\right]-f_{WS}^{RR}\left[n\right]|}{f_{ref}^{RR}\left[n\right]}$$

The averages of $MAE_{WS,i}$ and $MAE_{WS\%,i}$ over all subjects are denoted as $MAE_{WS}$ and $MAE_{WS\%}$, respectively.

A method specifically proposed to test the feasibility of a new measuring system for monitoring physiological parameters has been proposed in this study. Indeed, we performed Bland–Altman analysis [40] considering all the RR values collected by the proposed system (i.e., $f_{WS}^{RR}\left[n\right]$) and by the reference one (i.e., $f_{ref}^{RR}\left[n\right]$). This analysis was performed considering all the 8 volunteers in all the three scenarios. As recommended in [40], we computed the following parameters:

- $f_{RR}mean\left[n\right]$, calculated as the average value between $f_{ref}^{RR}\left[n\right]$ and $f_{WS}^{RR}\left[n\right]$;
- $\Delta f_{RR}$, calculated as the difference between $f_{ref}^{RR}\left[n\right]$ and $f_{WS}^{RR}\left[n\right]$;
- Mean of the differences (MOD), calculated as the mean of the difference between $f_{ref}^{RR}\left[n\right]$ and $f_{WS}^{RR}\left[n\right]$;
- Limits Of agreement (LOAs), calculated as $MOD\pm (1.96\cdot STD(\Delta f_{RR}$)).

### 5.2. Cardiac Activity: Data Analysis

According to Figure 1, to monitor the cardiac activity we considered the following signals:

- $s_{a_{x}}\left(t\right)$, which denotes the acceleration along x-axis of the M-IMU;
- $s_{g_{x}}\left(t\right)$ denoting the angular rotation around x-axis of the M-IMU;

Firstly, we band-pass filtered the two signals from 0.7 Hz to 20 Hz in order to remove or minimize bias, breathing activity-related signal and high frequency noise [41]. The choice of this filtering frequency band allowed preserving the informative content related to SCG [33,42]. Subsequently, in order to enhance the effect of the heart beat on recorded signals, we computed the Hilbert transform of $s_{a_{x}}\left(t\right)$ and $s_{g_{x}}\left(t\right)$. It is typically used in SCG and GCG data analysis [43], and given a generic signal s(t), its Hilbert transform is defined as follows:

(3) $$\hat{s}\left(t\right)=\frac{1}{\pi }\int_{-\infty }^{+\infty }\frac{s(\xi )}{t-\xi }\;d\xi ,$$

The outcome of the Hilbert transform, i.e., $\hat{s}\left(t\right)$, is a complex signal containing in its real part the copy of s(t) and in its imaginary part a 90 deg phase shift of s(t) itself. Assuming that the heart-beat activity (h(t)) is hidden and only its modulation can be measured, it is possible to model the recorded signal (s(t)) as follows [44]:

(4) $$s\left(t\right)=h\left(t\right)cos\left(2\pi f_{0}t\right)+\epsilon \left(t\right)$$

In (4), $cos(2\pi f_{0}t)$ denotes the modulating term [44], while ϵ(t) denotes additive noise. Therefore, according to the effect of (3) on the input signal, it is possible to extract h(t) as follows:

(5) $$h\left(t\right)=\sqrt{{\left(ℜ\left(\hat{s}\left(t\right)\right)\right)}^{2}+{\left(ℑ\left(\hat{s}\left(t\right)\right)\right)}^{2}},$$

denoting $ℜ(\hat{s}\left(t\right))$ and $ℑ(\hat{s}\left(t\right))$ the real part and the imaginary part of $\hat{s}\left(t\right)$, respectively.

To estimate HR we considered the following signals related to the *WS*:

- $h_{a_{x}}\left(t\right)$, denoting the heart-beat activity estimated considering $s_{a_{x}}\left(t\right)$;
- $h_{g_{x}}\left(t\right)$, denoting the heart-beat activity estimated considering $s_{g_{x}}\left(t\right)$;

All the above-mentioned signals were further filtered using a zero-phase shift band-pass filter from 0.7 to 5 Hz, in order to remove bias and obtain the heart-beat envelope (<5 Hz) [43].

The ECG signal recorded by the reference system and band-pass filtered from 0.7 to 20 Hz is denoted as $h_{ref}\left(t\right)$.

#### 5.2.1. Frequency Domain Analysis

For the *i*-th subject we computed the error between the HR estimated using the spectrum of $h_{ref}\left(t\right)$ ($F_{ref,i}^{HR}$) and $h_{WS}\left(t\right)$ ($F_{WS,i}^{HR}$) in each scenario as follows:

(6) $${\tilde{F}}_{WS,i}^{HR}=\left|F_{ref,i}^{HR}-F_{WS,i}^{HR}\right|$$

$F_{ref}^{HR}$ and $F_{WS}^{HR}$ correspond to the highest peaks in the spectra within the range 0.7–4 Hz of the signals collected by the reference system and the wearable device, respectively. Thus, $F_{WS}^{HR}$ was calculated by considering either $h_{a_{x}}$ or $h_{g_{x}}$. As for the RR analysis (described in Section 5.1.1), the spectra of the signals were computed by considering the power spectral density (PSD) using a Welch’s overlapped segment averaging estimator over the entire duration of the trials (180 s). In addition, we computed the percentage version of (6) as follows:

$${\tilde{F}}_{WS\%,i}^{HR}=\frac{|F_{ref,i}^{HR}-F_{WS,i}^{HR}|}{F_{ref,i}^{HR}}.$$

The averages of subjects of ${\tilde{F}}_{WS,i}^{HR}$ and ${\tilde{F}}_{WS\%,i}^{HR}$ are denoted as ${\tilde{F}}_{WS}^{HR}$ and ${\tilde{F}}_{WS,i}^{HR}$, respectively.

#### 5.2.2. Windowed Frequency Domain Analysis

To further investigate the HR estimation capabilities of the proposed *WS*, we implemented a new frequency domain analysis, considering 30 s lasting windows to compute the PSD.

To that end, we considered only $h_{g_{x}}\left(t\right)$, being the most reliable according to Section 6.2.2, and we computed its spectrum by using the the MATLAB^®^ "*pwelch*" function with a Hamming window of 30 s with an overlap between segments of 50%.

Similarly to (6), considering the *i*-th subject and the *k*-th window, we computed the error between the HR estimated using the spectrum of $h_{ref}\left(t\right)$ ($F_{ref,ik}^{HR}$) and $h_{g_{x}}\left(t\right)$ ($F_{g_{x},ik}^{HR}$) in each scenario as follows:

(7) $${\tilde{f}}_{g_{x},ik}^{HR}=\left|F_{ref,ik}^{HR}-F_{g_{x},ik}^{HR}\right|$$

In addition, we computed the percentage version of (7) as follows:

$${\tilde{f}}_{g_{x}\%,ik}^{HR}=\frac{|F_{ref,ik}^{HR}-F_{g_{x},ik}^{HR}|}{F_{ref,ik}^{HR}}$$

The averages of ${\tilde{f}}_{g_{x},ik}^{HR}$ and ${\tilde{f}}_{g_{x}\%,ik}^{HR}$ for windows are denoted as ${\tilde{f}}_{g_{x},i}^{HR}$ and ${\tilde{f}}_{g_{x}\%,i}^{HR}$, respectively; their averages for subjects are denoted as ${\tilde{f}}_{g_{x}}^{HR}$ and ${\tilde{f}}_{g_{x}\%}^{HR}$.

## 6. Results

### 6.1. Respiratory Activity

#### 6.1.1. Frequency Domain Analysis

**Figure 4:**
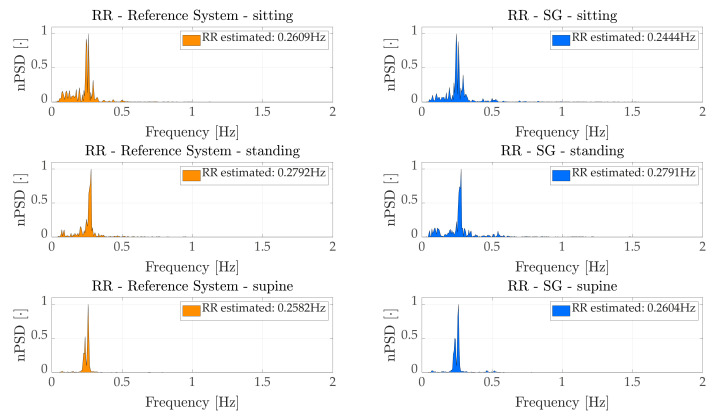
nPSD of $r_{ref}$ (left) and $r_{WS}$ (right) a representative subjects in each scenario.

The frequency domain analysis allowed us to estimate the average RR during the entire duration for each volunteer in each scenario (i.e., standing, sitting and supine). An example of a signal spectrum for a representative subject is presented in Figure 4 which shows the normalized PSD (nPSD), computed by dividing the amplitude of the spectrum by its maximum peak value, of both the reference system and the *WS* in all scenarios.

Table 2 and Table 3 report the values of ${\tilde{F}}_{WS,i}^{RR}$ and ${\tilde{F}}_{WS\%,i}^{RR}$ in the upper part and their average along subjects, i.e., ${\tilde{F}}_{WS}^{RR}$ and ${\tilde{F}}_{WS\%}^{RR}$, respectively, in the lower part in all three scenarios. The worst case is related to subject 8 during the scenario "supine," in which the system apparently failed in estimating the average RR. This might have been caused by a too low or absent pre-strain on the sensing elements due to the supine position. However, if such a value is discarded the average error in the supine scenario is equal to 0.14 bpm.

#### 6.1.2. Time Domain Analysis

**Figure 5:**
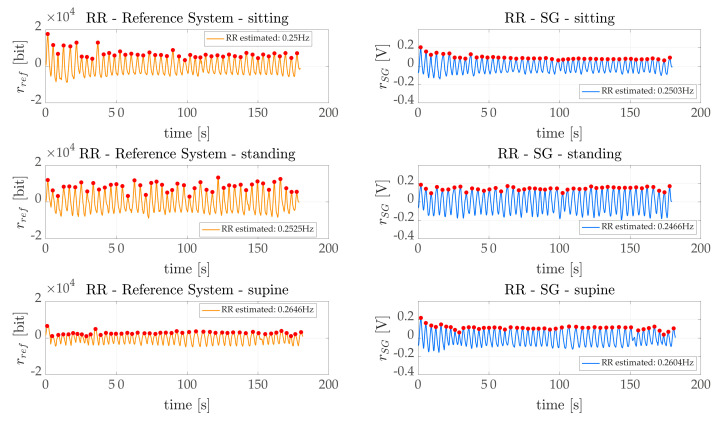
$r_{ref}$ (left) and $r_{WS}$ (right) plotted over time for all scenarios using a representative subject. Peaks selected using the method presented in Section 5 were superimposed on the signals (red circles).

The behaviours of $r_{ref}\left(t\right)$ and $r_{WS}\left(t\right)$ over time are reported in Figure 5 in all scenarios for a representative subject.

The values of $MAE_{WS,i}$ and $MAE_{WS\%,i}$ in all scenarios are reported in the upper part of Table 4 and Table 5, and their averages for subjects ($MAE_{WS}$ and $MAE_{WS\%}$) in the lower part.

**Figure 6:**
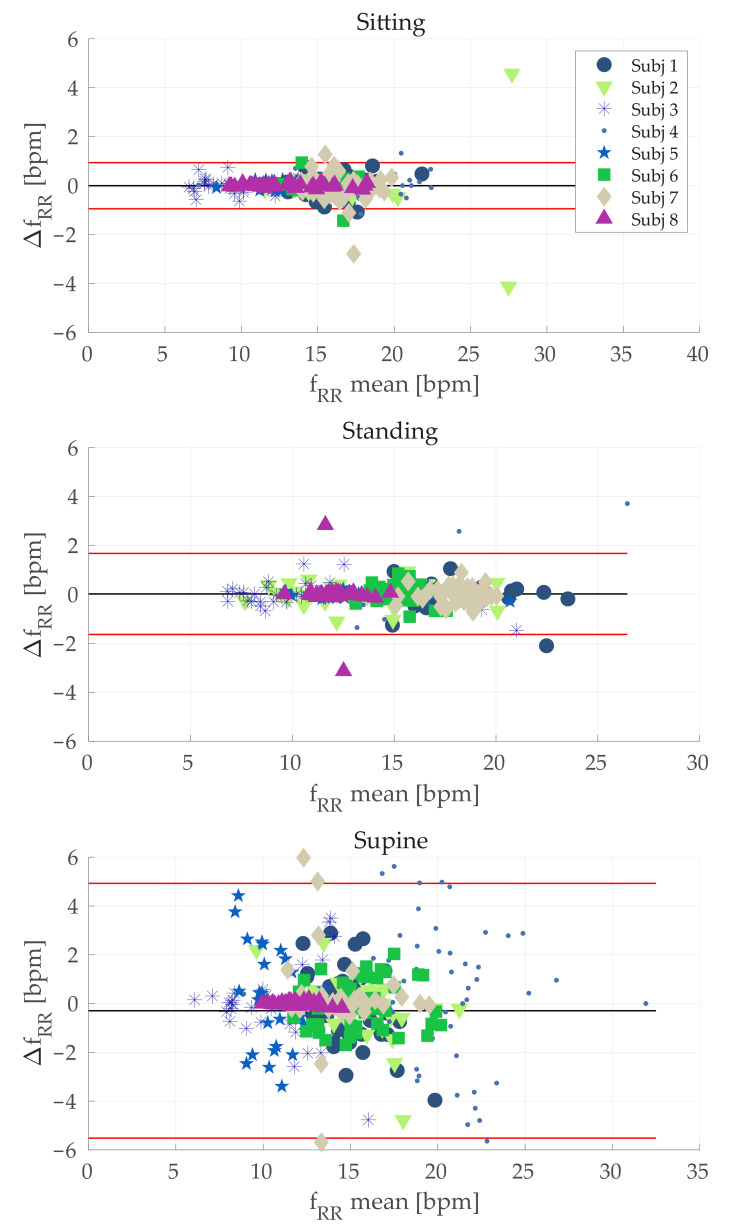
Plot related to Bland-Altman analysis for each scenario. Each plot contains all breath-by-breath RR values estimated for each subject. $\Delta f_{RR}=f_{ref}^{RR}\left[n\right]-f_{WS}^{RR}\left[n\right]$, and $f_{mean}^{RR}=\frac{f_{ref}^{RR}\left[n\right]+f_{WS}^{RR}\left[n\right]}{2}$, for *n*-th breath estimated.

Concerning the Bland–Altman analysis, the values of MODs and LOAs estimated for each scenario are reported in Table 6 and depicted in Figure 6.

### 6.2. Cardiac Activity

#### 6.2.1. Frequency Domain Analysis

According to Section 5, we have estimated HR considering $h_{a_{x}}\left(t\right)$ and $h_{g_{x}}\left(t\right)$. The best results were obtained considering $h_{g_{x}}\left(t\right)$, and the standing scenario was the worst case. The results related to ${\tilde{F}}_{WS,i}^{HR}$ and ${\tilde{F}}_{WS,i}^{HR\%}$ are reported in Table 7 and Table 8, respectively.

**Figure 7:**
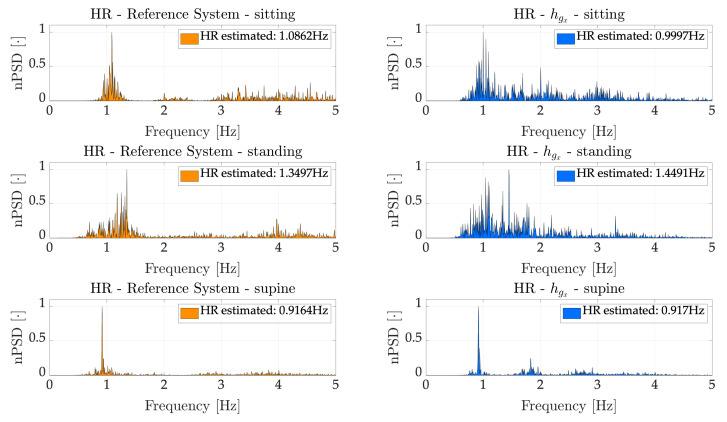
PSD of $h_{ref}$ (left) and $h_{g_{x}}$ (right) for a representative subject and all scenarios.

An example of the spectra obtained considering $h_{g_{x}}\left(t\right)$ is presented in Figure 7, which refers to a representative subject.

#### 6.2.2. Windowed Frequency Domain Analysis

The results obtained for ${\tilde{f}}_{g_{x},i}^{HR}$ (i.e., the error between HR estimated using $h_{ref}$ and $h_{g_{x}}$ for *i*-th subject averaged along the 30 s time windows considered to compute the spectra) and ${\tilde{f}}_{g_{x}}^{HR}$ (i.e., ${\tilde{f}}_{g_{x},i}^{HR}$ averaged along subject) are reported for each scenario in Table 9. Conversely, results related to ${\tilde{f}}_{g_{x}\%,i}^{HR}$ are reported in Table 10.

## 7. Discussion and Conclusions

In this study, we presented a prototype of an unobtrusive and multiparametric wearable system for continuous monitoring of RR and HR. The feasibility of the system has been assessed in different static positions (i.e., sitting, standing and supine), simulating clinical and remote/home monitoring scenarios, and an occupational setting—specifically, a computer worker sitting at a desk. Continuously monitoring those parameters can provide useful information on the health status of an individual, including insights on upcoming potentially critical conditions, and can improve workers’ conditions in terms of health, well-being and safety [1,2,3]. Indeed, although HR is a well established parameter for evaluating an individual’s critical critical state, RR is mostly neglected. Indeed, RR is directly affected by the effort made (e.g., physical activity, load handling), the surrounding environment and the psycho-physical state. Thus, a system capable of jointly monitoring breathing activity and cardiac activity may be beneficial to providing comprehensive assessments of the mentioned conditions [3,12,45].

As shown in Figure 1, the proposed wearable system embeds four conductive textiles sewed into two elastic bands located on the chest wall of the user (pulmonary rib cage and abdomen) for RR monitoring and an inertial measurement unit (IMU) integrated within a custom and compact PCB (located on the left side of pulmonary rib cage) for retrieving HR in terms of SCG and GCG.

Concerning the breathing activity, we monitored both average RR, by means of a frequency domain analysis (Section 5.1.1), and RR breath-by-breath, through a time domain analysis (Section 5.1.2). In both cases we considered $r_{sg}\left(t\right)$ band-pass filtered using a zero phase shift filter and we compared the estimated RR with the one estimated by the reference system ($r_{ref}\left(t\right)$). According to Section 6.1.1, the average RR estimated by the proposed *WS* can be considered as reliable, since the errors obtained were, on average, fractions of the breath-per-minute in sitting and standing tasks. The average error was ~3 bpm when considering the supine task, which corresponds to an average percentage error of ~9%. Such results were confirmed in the time domain analysis (Section 6.1.2). Indeed, the $MAE_{WS}$ obtained for sitting and standing are fractions of the breaths per minute. Additionally, in the worst case scenario (represented by the supine task) acceptable errors were obtained (~1.5 bpm, corresponding to a $MAE_{WS\%}$ of ~9.5%). The same behavior was obtained considering Bland–Altman analysis (see Table 6 and Figure 6). Although the MOD value was acceptable in all the cases, the LOAs were high especially for the supine position. A possible explanation for the worse results obtained during supine task may lie in an undesired interaction between the elastic bands with the support used let subjects lay down. Probably, in this configuration the elastic band stretched, thereby worsening the signals recorded by conductive sensors. The use of bands with the rear part being stiff instead of being elastic might solve this issue. However, this is just a speculation and future investigations will be devoted to study such aspects.

As regards HR, we considered the signals recorded by IMU. Firstly, we employed the Hilbert Transform to enhance the contribute of the heartbeat on the recorded signals, as already used in similar applications [43,44]. This technique allowed us to obtain the heartbeat envelope relative to the *x*-axis of the accelerometer ($h_{a_{x}}$) and the *x*-axis of the gyroscope ($h_{g_{x}}$). Considering such signals, we implemented a frequency domain analysis to estimate an average HR during the entire duration of the trials. Afterwards, we estimated the average HR on 30 s time windows to better assess the capabilities of the proposed device. In both cases, the results were compared with respect the ECG recorded by the reference system. Regarding the average HR, estimated considering the entire duration of the trials, $h_{g_{x}}$ and $h_{a_{x}}$ returned similar results in sitting and supine tasks, while $h_{g_{x}}$ (average error of 3.81 bpm, corresponding to a percentage error of 5.75%) prominently outperformed $h_{a_{x}}$ (average error of 13.82 bpm, corresponding to a percentage error of 23.26%) considering the standing task. This is likely due to the higher sensitivity of the accelerometer to the body motions, which are higher in standing being the subjects less constrained than in sitting and supine. As expected, the best results were obtained in the supine scenario (average error of 0.29 bpm and percentage error of 0.46% considering $h_{g_{x}}$, while 0.37 bpm and 0.59% considering $h_{a_{x}}$), where most of the movements detected by the IMU are due to heartbeat, once the respiration has been filtered out. Considering the average HR on a 30 s time window, we considered only $h_{g_{x}}$, on the basis the better results obtained in the above-mentioned frequency domain analysis, which allowed obtaining error (averaged along subjects) of ~3.5 bpm (~5.5%), ~4.5 bpm (~3.2%) and ~16 bpm (~18.9%) in sitting, supine and standing tasks respectively.

A few studies have investigated to simultaneously monitor breathing and cardiac activities, and the proposed system show error in line with the systems reported in literature [22,36,37,38]. Results presented in [22] show errors smaller than ~2% and ~6%; however, fiber Bragg grating sensors were used, which require more expensive and bulky systems to retrieve the signals, and above all, the HR were estimated during apnea. In [36], where the authors used a belt embedding textile electrodes for recording ECG and breathing activity through impedance variation of the chest wall. They showed errors of ~2% concerning RR estimation and better results in terms of HR estimation. Despite the very good results obtained, the main drawback of this solution lays in the contact required between the electrodes and the skin of the subjects and the need to continue guarantee a low impedance at the contact points. A similar approach was proposed by [37]; however, no performance comparisons with a reference system were presented. In [38] the system proposed is based on a single piezoelectric sensors which allowed the authors to obtain errors of (in average) ~10% and ~6% for RR and HR respectively. To conclude the comparison, the main advantage of our solution lays in the ease of use, simple and low cost electronics required and high wearability and comfort, which does not require direct contact with the skin or further adjustments of the sensors after they are worn by the user. Moreover, because of the presence of IMU within the device, it is possible to exploit their sensitivity on body motion artifacts to further improve the estimation of RR, similarly to [20,46].

The present work is mainly focused on the design of the device and the technology, rather than implementing or assessing robust and efficient algorithms to remove motion artifact from recorded signals during the everyday life. However, we reckon that the problem of motion artifact removal should be taken into account since in a real-life scenario, movements of the subjects can occur. To overcome this concern, different solutions have been proposed for both RR [20,28,47,48] and regarding HR [33,41,49,50,51]. Future works will be devoted to defining a tailored approach on our system, by combining the two different sensor technologies embedded (i.e., textile strain sensors and an M-IMU) to develop a sensor-fusion algorithm to remove motion artifacts occurring during real life.

Future works will be devoted to further improve the proposed system to enhance its capabilities of HR and RR estimation. Indeed, in its present version, the sensors are sewed on two elastic bands which, despite being comfortable for an ease removal, introduce an additional compliance thus reducing the sensitivity of the conductive sensors. This contingency does not allow them to reliably catch the SCG activity. Therefore, as a future work we are planning on sewing the sensors directly onto an elastic t-shirt (i.e., sportswear) to reduce as much as possible additional compliant elements between the sensors and the user, aiming at investigating whether conductive sensors allow also reliably and robustly monitoring HR, as much as they do with respect RR. Moreover, this may lead to a lower system complexity, to slightly improve its cost and to provide a more comfortable system. In addition, we will test the improved system on a larger population, including females and pathological subjects, to evaluate its potential use also in clinical settings. Since we tested the device on only male subjects, we can just speculate that the use of the proposed device might not be of any discomfort on female subjects. Taking into account what most women wear during sport activities (i.e., sport bras), the use of the upper band of the proposed device should not be of relevant discomfort, since they also allow being regulated in length thanks to the provided Velcro. We are convinced that with the improved device this potential discomfort will be avoided. In addition, the respiratory movements will be hardly detected by the upper band due to the presence of the breast. However, this hurdle will be overcome thanks to the presence of the second band. Moreover, we will evaluate the system performances during different daily living activities which result to be more challenging but represent a typical use of the proposed system. Finally, it is worth noting that we used basic data analysis techniques; therefore, more sophisticated analyses (e.g., based on machine learning methods) may allow improving the estimation of respiratory and cardiac parameters.

## Figures and Tables

**Table 1:** Age, body mass, height and body mass index (BMI) of the 8 volunteers.

Volunteer	Age [Years]	Body Mass [kg]	Height [m]	BMI [kg·m^−2^]
V1	30	72	1.69	25.2
V2	28	76	1.80	23.5
V3	26	88	1.89	24.6
V4	27	70	1.75	22.9
V5	28	74	1.75	24.2
V6	25	98	1.77	31.9
V7	25	63	1.65	23.1
V8	33	62	1.63	23.3

**Table 2:** The absolute error of RR estimated for each volunteer, considering the three scenarios. The average value of the mentioned error for every subject is also shown.

Volunteer	${\tilde{F}}_{\mathrm{WS},i}^{\mathrm{RR}}\;\left[\mathrm{bpm}\right]$-Sitting	${\tilde{F}}_{\mathrm{WS},i}^{\mathrm{RR}}\;\left[\mathrm{bpm}\right]$-Standing	${\tilde{F}}_{\mathrm{WS},i}^{\mathrm{RR}}\;\left[\mathrm{bpm}\right]$-Supine
1	0.99	0.01	0.01
2	0.06	2.39	0.36
3	0.07	0.06	0.01
4	0.02	0.01	0.03
5	0.04	0.00	0.13
6	0.01	0.01	0.01
7	0.11	0.27	0.34
8	0.04	0.01	22.57
Average	0.17	0.35	2.95

**Table 3:** The percentage of absolute error of RR estimated for each volunteer, considering the three scenarios. The average value of the mentioned error for each of the subjects is also shown.

Volunteer	${\tilde{F}}_{\mathrm{WS}\%,i}^{\mathrm{RR}}\;\left[\%\right]$-Sitting	${\tilde{F}}_{\mathrm{WS}\%,i}^{\mathrm{RR}}\;\left[\%\right]$-Standing	${\tilde{F}}_{\mathrm{WS}\%,i}^{\mathrm{RR}}\;\left[\%\right]$-Supine
1	6.78	0.05	0.87
2	0.40	25.43	2.16
3	0.95	0.72	0.08
4	0.09	0.10	0.13
5	0.33	0.01	1.19
6	0.06	0.08	0.04
7	0.72	1.47	2.58
8	0.32	0.07	66.94
Average	1.21	3.49	9.25

**Table 4:** $MAE_{WS}$ of RR estimated for each volunteer, considering the three scenarios. The average value of the mentioned error for each subject is also shown.

Volunteer	${\mathrm{MAE}}_{\mathrm{WS},i}\;\left[\mathrm{bpm}\right]$-Sitting	${\mathrm{MAE}}_{\mathrm{WS},i}\;\left[\mathrm{bpm}\right]$-Standing	${\mathrm{MAE}}_{\mathrm{WS},i}\;\left[\mathrm{bpm}\right]$-Supine
1	0.24	0.61	1.16
2	0.30	0.28	1.08
3	0.22	0.39	1.11
4	0.24	0.33	3.14
5	0.11	0.11	1.92
6	0.18	0.25	0.87
7	0.36	0.23	1.78
8	0.05	0.23	0.07
Average	0.21	0.30	1.39

**Table 5:** $MAE_{WS\%}$ of RR estimated for each volunteer, considering the three scenarios. The average value of the mentioned error for each subject is also shown.

Volunteer	${\mathrm{MAE}}_{\mathrm{WS}\%,i}\;\left[\%\right]$-Sitting	${\mathrm{MAE}}_{\mathrm{WS}\%,i}\;\left[\%\right]$-Standing	${\mathrm{MAE}}_{\mathrm{WS}\%,i}\;\left[\%\right]$-Supine
1	1.52	3.17	7.72
2	1.44	2.25	7.41
3	2.56	3.59	9.08
4	1.36	1.89	15.00
5	0.91	0.81	18.09
6	1.15	1.64	5.65
7	2.25	1.29	12.57
8	0.37	1.93	0.57
Average	1.45	2.07	9.51

**Table 6:** Results of the Bland–Altman analysis of speed for the three tested scenarios.

Scenario	MOD [bpm]	LOA-Upper [bpm]	LOA-Lower [bpm]
Sitting	−0.0039	0.9391	−0.9470
Standing	0.0186	1.6753	−1.6392
Supine	−0.2948	4.9264	−5.5160

**Table 7:** Error between average HR estimated using $r_{ref}$ and $r_{WS}$ from $h_{a_{x}}\left(t\right)$ and $h_{g_{x}}\left(t\right)$ signals over subjects (${\tilde{F}}_{WS,i}^{HR}$) for each scenario.

Volunteer	${\tilde{F}}_{\mathrm{WS},i}^{\mathrm{HR}}\;\left[\mathrm{bpm}\right]$-Sitting	${\tilde{F}}_{\mathrm{WS},i}^{\mathrm{HR}}\;\left[\mathrm{bpm}\right]$-Standing	${\tilde{F}}_{\mathrm{WS},i}^{\mathrm{HR}}\;\left[\mathrm{bpm}\right]$-Supine
$h_{g_{x}}\left(t\right)$			
1	0.56	2.92	0.89
2	3.83	0.05	0.18
3	5.19	5.97	0.03
4	0.28	13.07	0.09
5	0.13	1.98	0.06
6	0.30	0.23	0.28
7	0.18	6.20	0.02
8	0.25	0.08	0.80
Average	1.34	3.81	0.29
$h_{a_{x}}\left(t\right)$			
1	0.41	32.80	0.06
2	3.83	0.05	0.18
3	8.45	24.55	0.03
4	6.29	0.73	2.23
5	0.13	0.02	0.06
6	3.04	9.93	0.28
7	0.16	36.21	0.02
8	0.25	6.24	0.12
Average	2.82	13.82	0.37

**Table 8:** Percentage of error between average HR estimated using $r_{ref}$ and $r_{WS}$ from $h_{a_{x}}\left(t\right)$ and $h_{g_{x}}\left(t\right)$ signals over subjects (${\tilde{F}}_{WS\%,i}^{HR}$) for each scenario.

Volunteer	${\tilde{F}}_{\mathrm{WS}\%,i}^{\mathrm{HR}}\;\left[\%\right]$-Sitting	${\tilde{F}}_{\mathrm{WS}\%,i}^{\mathrm{HR}}\;\left[\%\right]$-Standing	${\tilde{F}}_{\mathrm{WS}\%,i}^{\mathrm{HR}}\;\left[\%\right]$-Supine
$h_{g_{x}}\left(t\right)$			
1	0.83	6.44	1.53
2	5.54	0.06	0.28
3	7.96	7.37	0.06
4	0.41	17.98	0.14
5	0.21	2.69	0.10
6	0.35	0.23	0.37
7	0.31	11.21	0.04
8	0.30	0.09	1.14
Average	1.99	5.75	0.46
$h_{a_{x}}\left(t\right)$			
1	0.61	72.34	0.11
2	5.54	0.06	0.28
3	12.96	30.31	0.06
4	9.16	1.00	3.59
5	0.21	0.03	0.10
6	3.53	9.85	0.37
7	0.28	65.49	0.04
8	0.30	7.01	0.17
Average	4.07	23.26	0.59

**Table 9:** Values of ${\tilde{f}}_{g_{x},i}^{HR}$ obtained for all subjects in the three experimental scenarios.

Volunteer	${\tilde{f}}_{g_{x},i}^{\mathrm{HR}}\;\left[\mathrm{bpm}\right]$-Sitting	${\tilde{f}}_{g_{x},i}^{\mathrm{HR}}\;\left[\mathrm{bpm}\right]$-Standing	${\tilde{f}}_{g_{x},i}^{\mathrm{HR}}\;\left[\mathrm{bpm}\right]$-Supine
1	5.45	27.27	0.18
2	0.00	27.45	0.67
3	13.45	16.18	1.00
4	8.60	4.60	5.20
5	0.20	0.00	0.00
6	0.20	35.60	28.73
7	0.80	15.20	0.00
8	0.20	0.20	0.40
Average	3.61	15.81	4.52

**Table 10:** Values of ${\tilde{f}}_{g_{x}\%,i}^{HR}$ obtained for all subjects in the three experimental scenarios.

Volunteer	${\tilde{f}}_{g_{x}\%,i}^{\mathrm{HR}}\;\left[\%\right]$-Sitting	${\tilde{f}}_{g_{x}\%,i}^{\mathrm{HR}}\;\left[\%\right]$-Standing	${\tilde{f}}_{g_{x}\%,i}^{\mathrm{HR}}\;\left[\%\right]$-Supine
1	8.02	53.99	0.29
2	0.00	16.99	1.08
3	20.96	20.91	1.98
4	13.03	6.33	8.01
5	0.31	0.00	0.00
6	0.23	30.01	13.42
7	1.30	22.56	0.00
8	0.29	0.24	0.58
Average	5.52	18.88	3.17

## Data Availability

The data presented in this study are available on request from the corresponding author. The data are not publicly available due to privacy reason.

## Abbreviations

The following abbreviations are used in this manuscript:

WS	Wearable System
RR	Respiratory Rate
HR	Heart Rate
PSD	Power Spectral Density
nPSD	Normalized Power Spectral Density
PCB	Printed Circuit Board
ADC	Analog Digital Converter
M-IMU	Magneto-Inertial Measurement Unit
IMU	Inertial Measurement Unit
BMI	Body Mass Index
RMS	Root Mean Square
MOD	Mean of Difference
LOA	Limit of Agreement
ECG	Electrocardiography
SCG	Seismocardiography
GCG	Gyrocardiography
